# Cerebral visual impairment

**Published:** 2024-02-09

**Authors:** Richard Bowman, P Vijayalakshmi

**Affiliations:** 1 Honorary Clinical Consultant: International Centre for Eye Health, London School of Hygiene & Tropical Medicine, London, UK.; 2 Senior Paediatric Ophthalmologist and Chief of Vision Rehabilitation Centre: Aravind Eye Care System, Madurai, India.

**Children with cerebral visual impairment can be helped to make the most of their vision, but early intervention is vital.**

**Figure:**
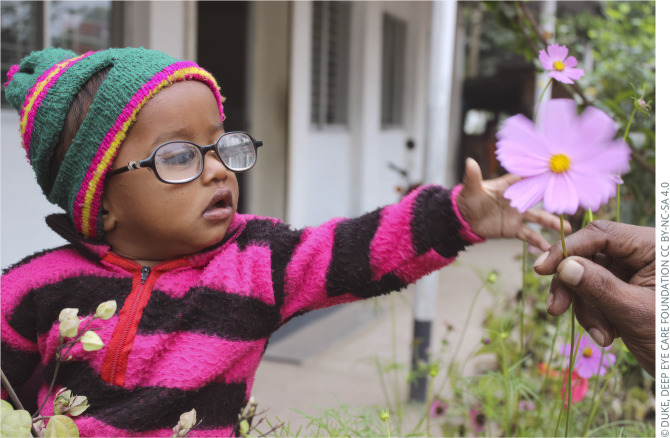
Children with cerebral visual impairment should have their refractive errors corrected. They can be taught ‘active looking’ by increasing their awareness of their hands and feet and encouraging them to reach out and touch the objects they are trying to look at. bangladesh

Cerebral vision impairment (previously called cortical vision impairment) includes all visual problems caused by brain damage.^1^ Children with cerebral palsy, due to damage to the parts of the brain which control movement, can also have cerebral vision impairment.

In cerebral vision impairment, the eyes can be completely healthy, but children struggle with one or more of the following:

- Seeing clearly (due to poor visual acuity)
- Recognising people and things which are familiar
- Difficulty going down steps or stairs due to lower visual field defects
- Functioning in a cluttered or visually ‘busy’ environment (e.g., struggling to find objects)
- Coordinating movements that depend on vision (e.g., picking up a small object).

If parents and teachers do not recognise that the child's challenges are due to a problem with their brain, they may think that the child is being uncooperative, disobedient, or clumsy.

Cerebral vision impairment is increasing in importance in low- and middle-income countries. This is because prematurity is an important cause of cerebral vision impairment, and – as a result of improvements in neonatal care in many low- and middle-income countries – premature babies are now surviving in greater numbers.

Brain damage associated with cerebral vision impairment cannot usually be treated. However, families can be supported to make changes in their home or how they interact with their child, based on their child's unique combination of difficulties, and this can improve the quality of their child's life.

Early intervention is vital – the younger a child is, the more their brain will be able to adapt and make good use of the support they receive.

## How does cerebral visual impairment affect children?

Cerebral visual impairment can cause any combination of the following, to a greater or lesser degree:

- Loss of visual acuity
- Falling over objects or difficulty going downstairs due to lower visual field defects
- Visual perception problems, such as difficulties moving around in cluttered or changing environments or not being able to find their way alone along a familiar route.

It is important to understand the wide variety of difficulties children with cerebral vision impairment can have, as coping strategies need to be tailored to the unique combination of difficulties experienced by each child.

## What causes cerebral vision impairment?

Children are either born with cerebral vision impairment, or it occurs during the first few years of life, following brain injury from a range of causes (Table 1).^2^ Cerebral vision impairment is common amongst children with cerebral palsy and special educational needs, such as cognitive/intellectual disabilities and/or autism.^3^

**Table 1:** Causes of cerebral vision impairment in high- and low-income settings

Cause	Low income**	High income
Prematurity	+++++	++++++++++
Neonatal sepsis	++++	+++
Birth asphyxia	++++	++
Meningitis	++	+
Neonatal jaundice	++++	+ / -
Cerebral malaria	++	-
Genetic conditions	-	+

** Limited data are available.

## What to look for at the local/primary level and when to refer

The following children must be **referred to the nearest eye centre where there is a paediatric ophthalmologist**, particularly if they have never been examined by one before:

1. All children with **cerebral palsy**. Some of the difficulties children with cerebral palsy have with everyday tasks or movement can be due to problems with their vision, not the cerebral palsy. Parents may not realise this, as their child's eyes appear normal.
2. Children with special educational needs, such as **cognitive/intellectual disabilities and/or autism**. Cerebral visual impairment can exacerbate these children's difficulties, as good vision is needed for normal intellectual and social development. Parents and health care personnel may attribute signs of cognitive visual impairment to the child's other condition.
3. All children who were **born very prematurely or who have had difficult births**, particularly if they were deprived of oxygen during delivery (birth asphyxia); these children are at high risk of cerebral palsy and cerebral visual impairment.
4. Children with a history of **neonatal sepsis**, **neonatal jaundice**, and/or **cerebral malaria** (see Table 1).
5. Otherwise healthy children, **if their parents or carers say that they have noticed the problems outlined on page 20 once their child is old enough to walk around**. The child may fall over things on the floor, struggle to see things that are moving quickly (like birds or animals), struggle to recognise people they know, or struggle to find their way to school on their own – even though they have travelled there several times before.

## What care do these children need?

Children who may have cerebral vision impairment must undergo an assessment of their **visual functioning** (visual acuity and visual fields), and an assessment to determine whether they have any problems with processing vision (visual perception).

Specialists can then explain to parents how they can support their child to make the best use of the vision they have, which will help their development.

## Diagnosis at the tertiary level

Cerebral vision impairment is initially diagnosed clinically, after ruling out eye conditions that could be responsible for poor vision. The diagnosis can sometimes be supported by a computed tomography (CT) or magnetic resonance imaging (MRI) scan, where available.

All children with developmental delays and/or cerebral palsy should be assessed.

1. Start by asking whether there is a history of any of the conditions which can cause brain damage in children (Table 1).
2. Ask whether the parents/carers have noticed any problems which they think might be due to their child not seeing normally.
3. Ask about visual milestones (see Figure 2, page 16) and test whether the child has reached the milestone appropriate for their age. For example, for a 9-month-old, put a small, bright object in front of them to see if they look at it and/or try to reach for it.
4. Measure visual acuity using age- and ability-specific tests, if possible.
5. Carry out refraction; cycloplegia may be needed.
6. Assess other visual functions, i.e., visual field, contrast sensitivity, colour vision, and depth perception.
7. Assess eye movements.
8. Check pupil reactions, the clarity of the ocular media, and the optic discs.

For children who are old enough to walk, ask the parents/carers a series of questions to assess the child's higher visual processing:

1. Does your child have difficulty going down steps or stairs?
2. Does your child have difficulty seeing things which are moving quickly, such as a bird or small animal, or a ball that is thrown?
3. Does your child have difficulty seeing something which is pointed out to them in the distance?
4. Does your child have difficulty locating an item of clothing in a pile of clothes?
5. Does your child find drawing or copying letters or words difficult and time-consuming?

If the parent/carer says yes to more than three of these questions, it is likely that the child has visual perception problems.

More specialised tests of visual function, which need to take into account the child's age, ability and level of cooperation, include testing their ability to:

- Detect small objects
- Discriminate two spatially separated targets (such as Lea paddles)
- Fixate on and follow a moving target
- Discriminate shape, size, and colour.

## Management at the tertiary level

A key principle in managing visual and neurodevelopmental problems in children is the importance of **early intervention**. This is because the young brain can change more readily than an older brain (i.e., is has more ‘plasticity’).

**“A key principle in managing visual and neurodevelopmental problems in children is the importance of early intervention.”**

As with any other child, significant refractive errors should be corrected. Children with cerebral palsy and other conditions of the brain often have poor accommodation. Spectacles with a near add can be very helpful even in young children, as good near vision is important for interacting and bonding with their mother.^4^ Yoked prisms (base-down prisms in front of both eyes) are useful for managing lower visual field defects.

Children can also be helped to make the most of the vision they have, by providing ‘active visual stimulation’.^4^ The basis of this approach is that vision is the sense which coordinates other sensory input. Without vision, or with poor vision, it is harder for children to make sense of their environment and to learn. Children can be taught ‘active looking’ by increasing their awareness of their hands and feet and encouraging them to reach out and touch the objects they are trying to look at. Carers must provide verbal explanations of what the child is experiencing in terms of what they are touching, tasting, or hearing to enable then to gain a better understanding of the world around them.

Another approach, which shows promise, is to tailor what can be done to address the specific problems of an individual child. For example, if a child cannot find something amongst a group of other items, make the visual environment simpler by removing any unnecessary items.

Difficulty going down steps may be due to a lower field defect and/or poor visual-motor coordination of the feet (in addition to any motor problems with the feet/legs). Strategies might include wearing brightly coloured shoes, feeling for the edge of the step with the feet, or using a stick to provide sensory contact with the ground. If a child cannot recognise a parent amongst a group of other people, the parent could wear an item of clothing of an agreed colour.^5^,^6^ A list of possible interventions can be found at tinyurl.com/cviguide. The CVI Scotland website (https://cviscotland.org/) has a lot of helpful information including videos and animations which help us to experience what it might be like to have some of the visual difficulties experienced by children with cerebral vision impairment.

## Counselling parents and carers

All parents/carers of children with cerebral visual impairment must be given a clear, understandable explanation of what is wrong and why this might have happened. Knowing that their child has a physical problem with seeing or understanding what they see can make parents realise that their child is not being naughty, difficult, or disobedient. This can change attitudes and relationships. Where possible, teach parents how to adapt to the needs of the child, as described in the previous section.

**Figure:**
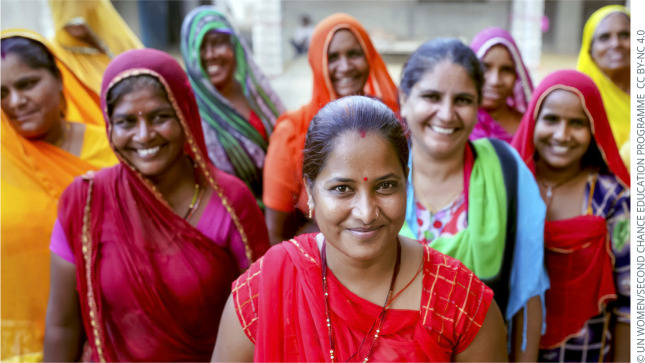
Agreeing a colour to wear (red) will help a child with cerebral vision impairment to find their mother in a crowd of women. india

## WORLD HEALTH ORGANIZATION

### New World Health Organization guidelines

**WHO has published two new documents relevant to child eye care.**

The World Health Organization (WHO) standards for improving the quality of care for small and sick newborns in health facilities has been updated; it now recommends identifying, screening and treating infants at risk of retinopathy of prematurity (ROP) as an evidence-based practice to improve the quality of intensive neonatal care. You can use this recommendation to advocate with ministries of health for more services for ROP in your country.

Quality statement 1.33 (new) states: **Small and sick newborns at risk of retinopathy of prematurity are appropriately identified, screened and treated.**

Download the document from tinyurl.com/WHOrop

WHO recommends that all newborns have their eyes screened as part of the general newborn assessment, to detect abnormalities such as cataract and retinoblastoma. This recommendation can be used to advocate with ministries of health to ensure eye screening is included in their child health programmes.

Recommendation 26 states: **Universal newborn screening for abnormalities of the eye is recommended and should be accompanied by diagnostic and management services for children identified with an abnormality.**

Download the document from tinyurl.com/WHOmat-care

### Important messages for eye health in babies and young children

Sharing the information in this poster with parents and carers can help to prevent life-long visual impairment and blindness in babies and young children. If you can, download the poster from our website (www.cehjournal.org), print it in colour, and display it at your local primary care facility.

**Have you noticed something white in the eye or eyes of your child?**

If so, tell the staff in the clinic

**Does your child have a squint?**

Tell the staff

**Does your child have red, sticky, or watering eyes?**

Tell staff in the clinic

**Is your child's eye painful, or you are worried they might not see normally?**

If you have noticed anything wrong with your child's eye or eyes, tell the staff in the clinic

**How you can prevent eye disease and keep your child's eyes healthy**
